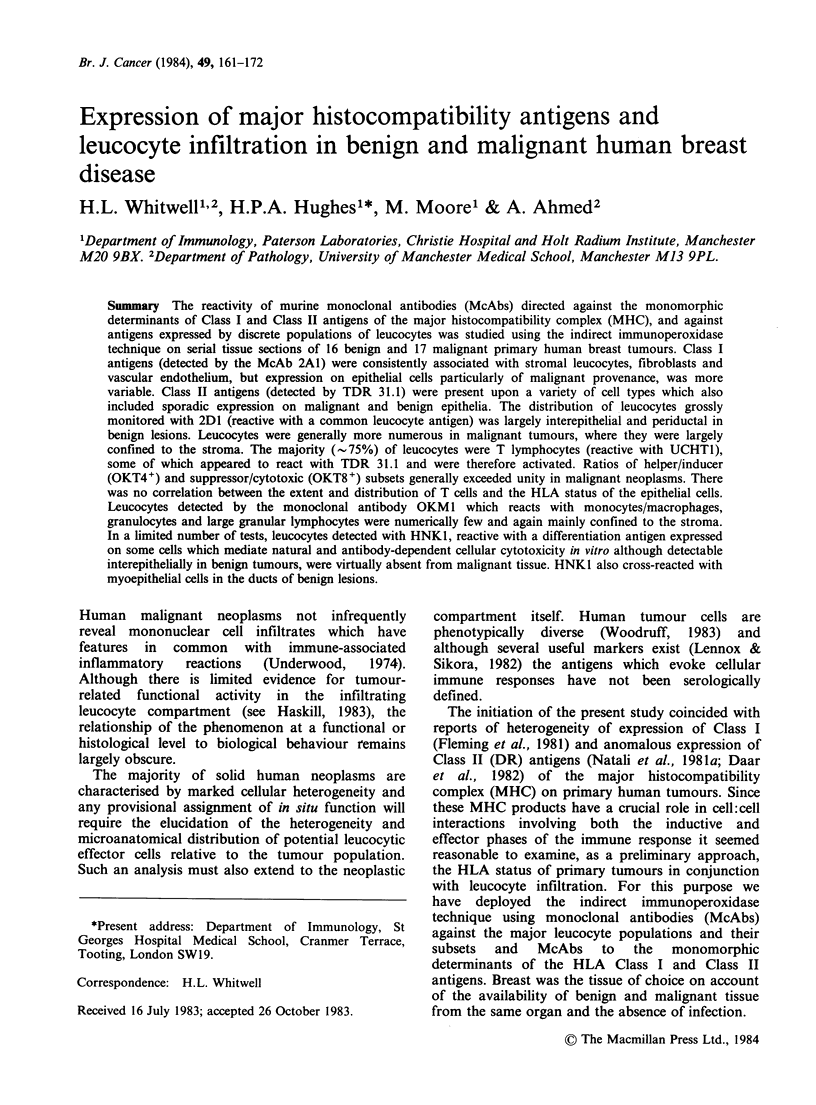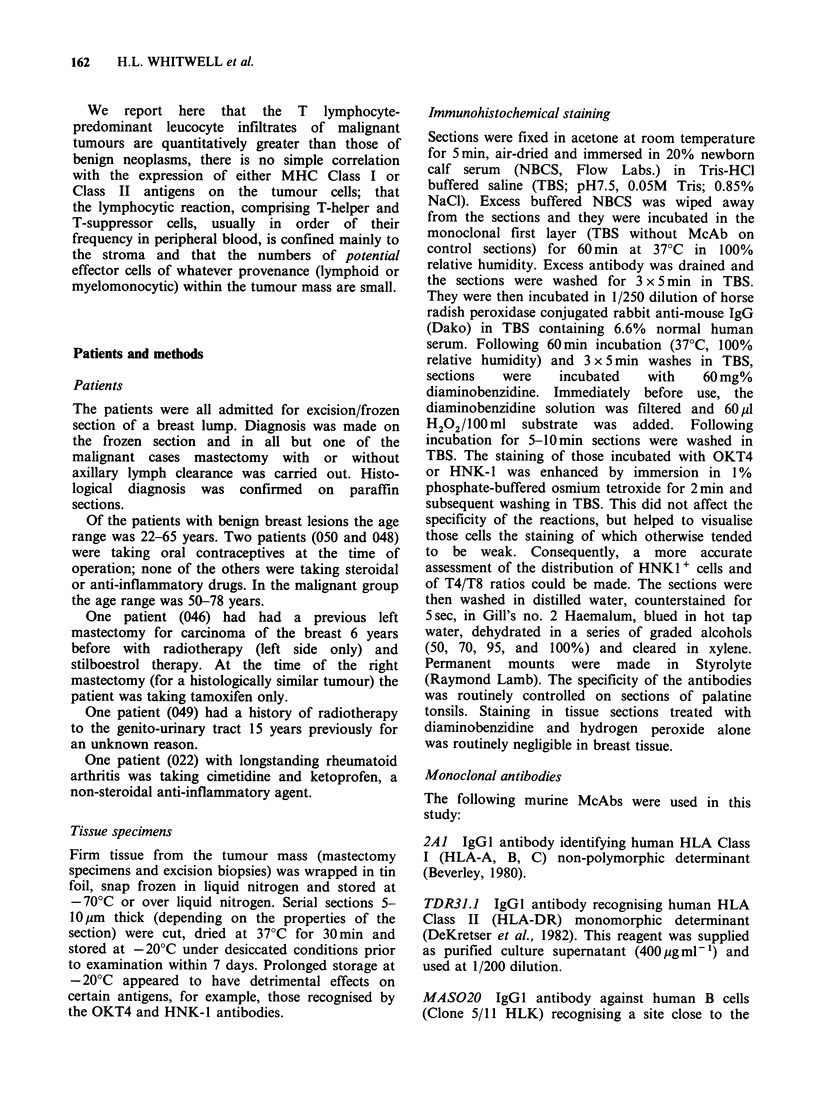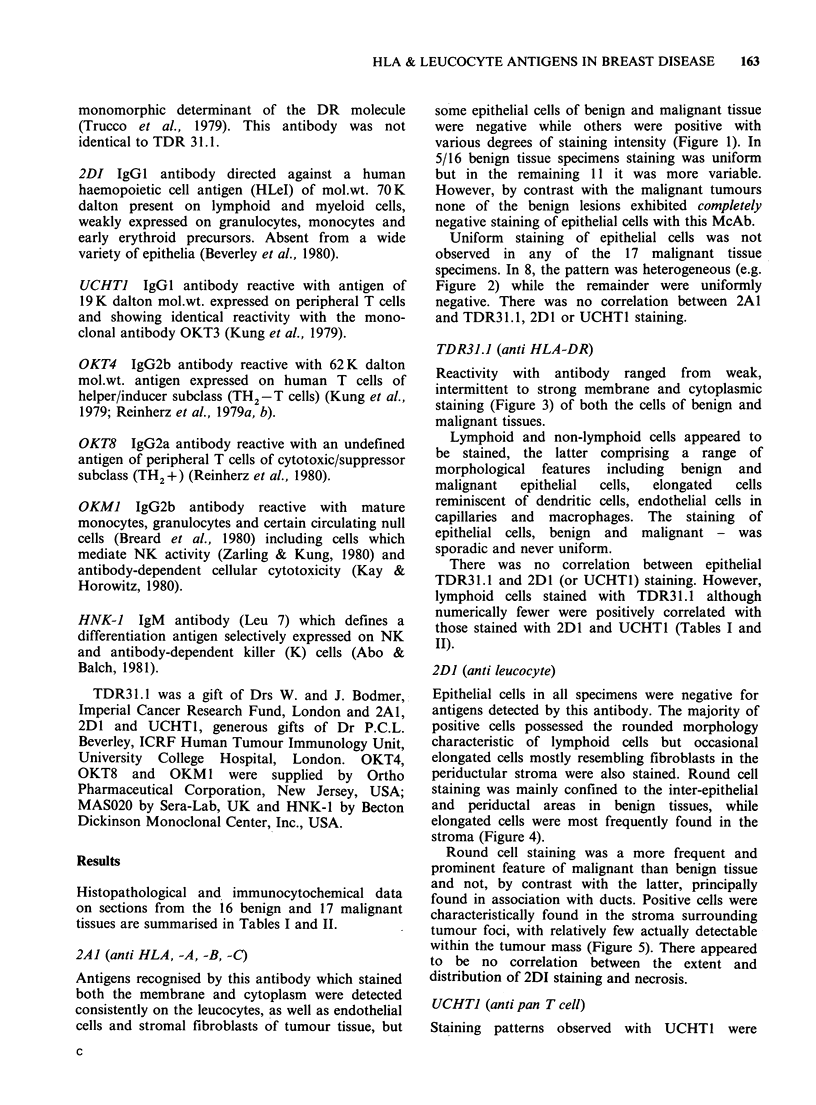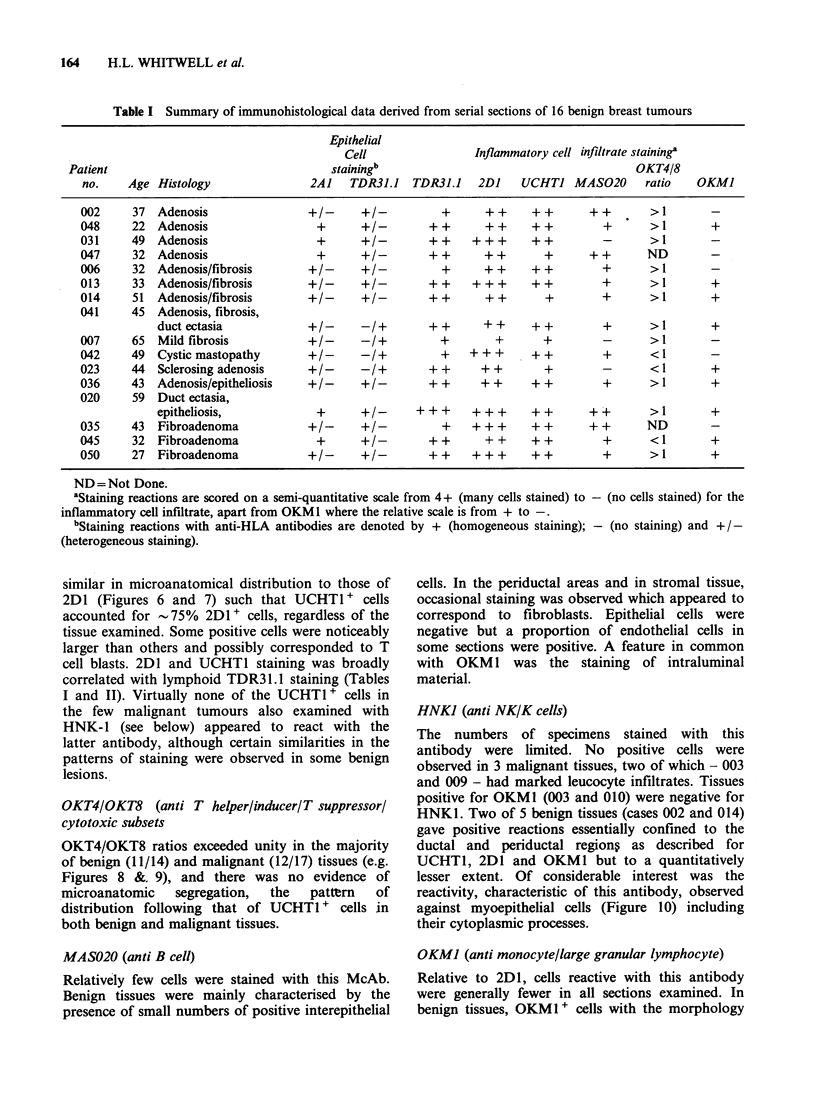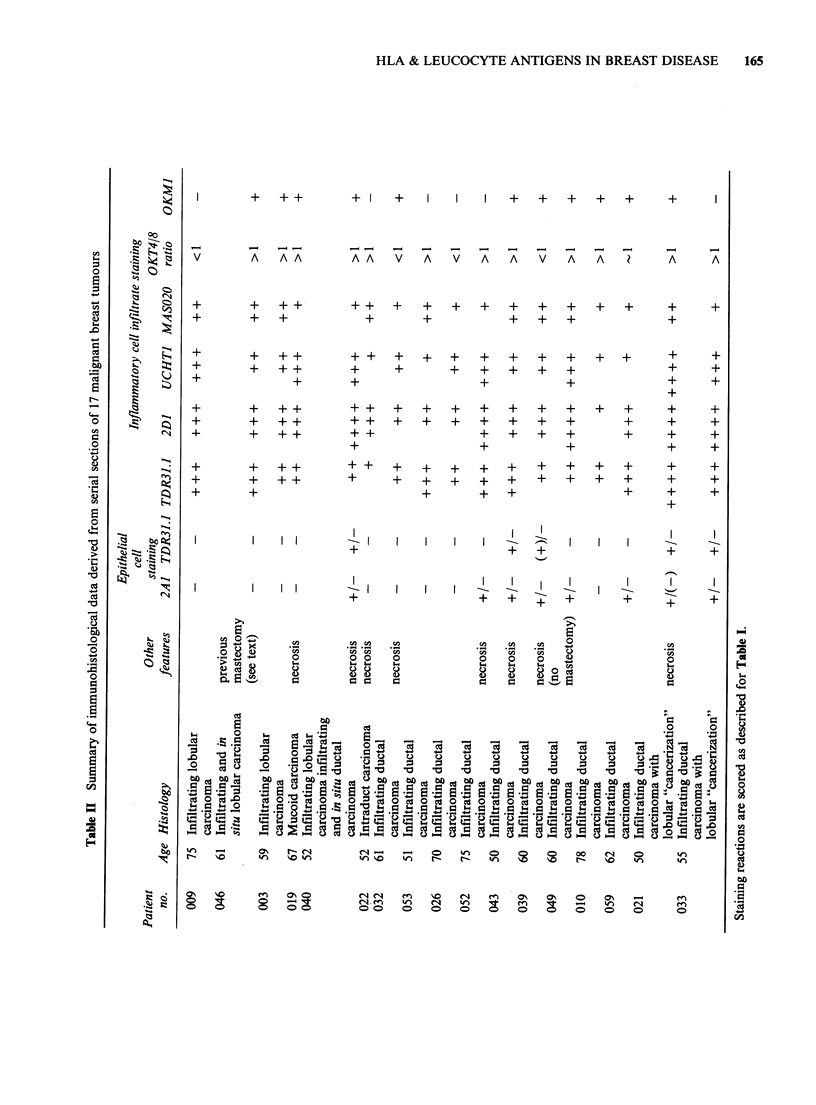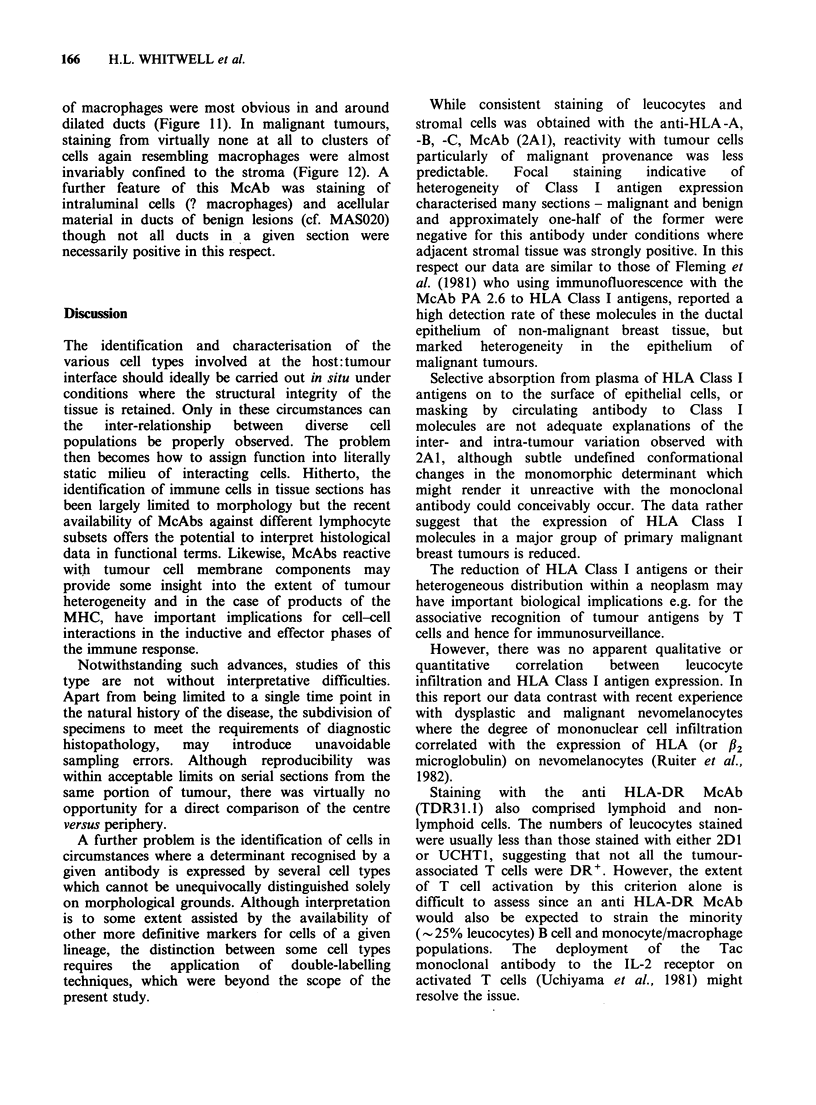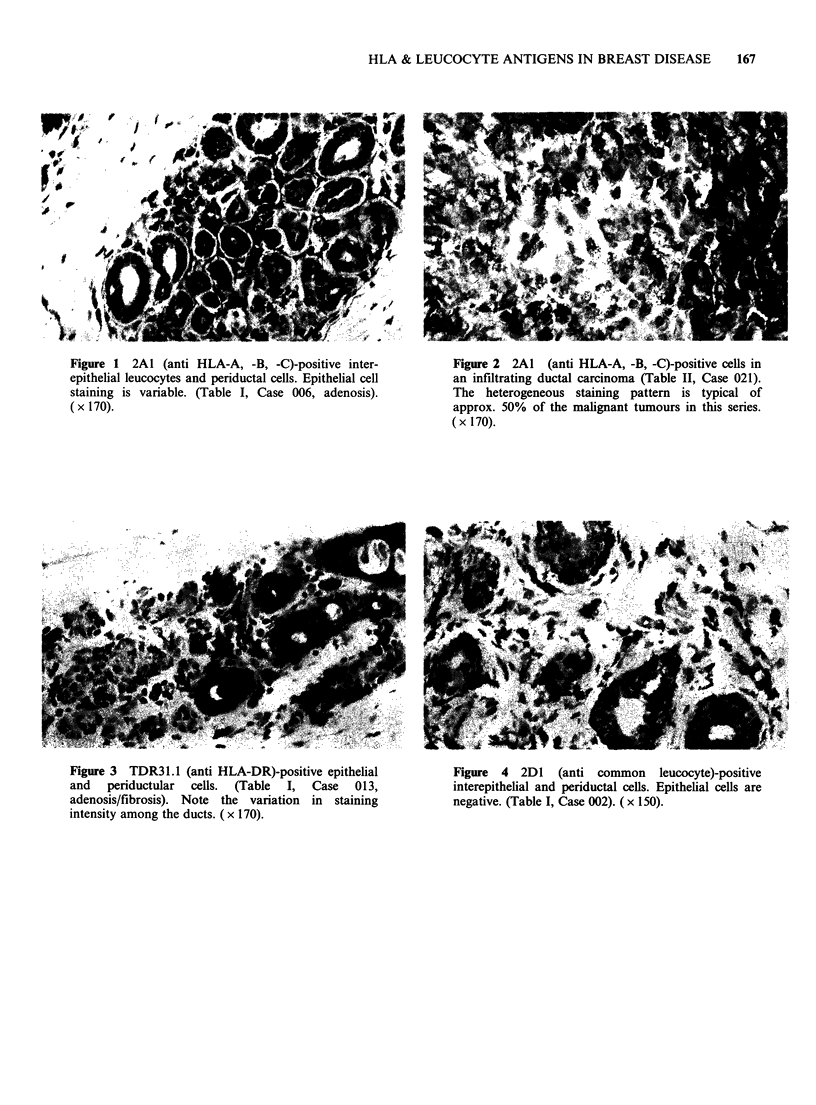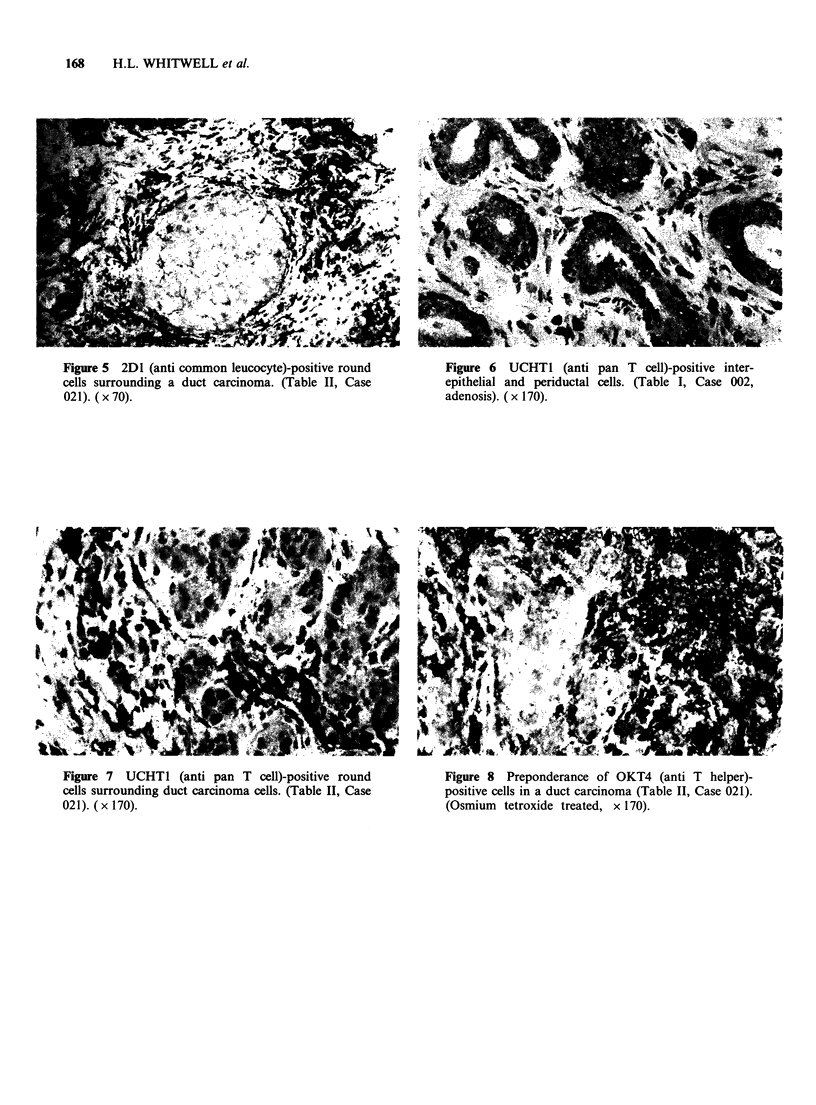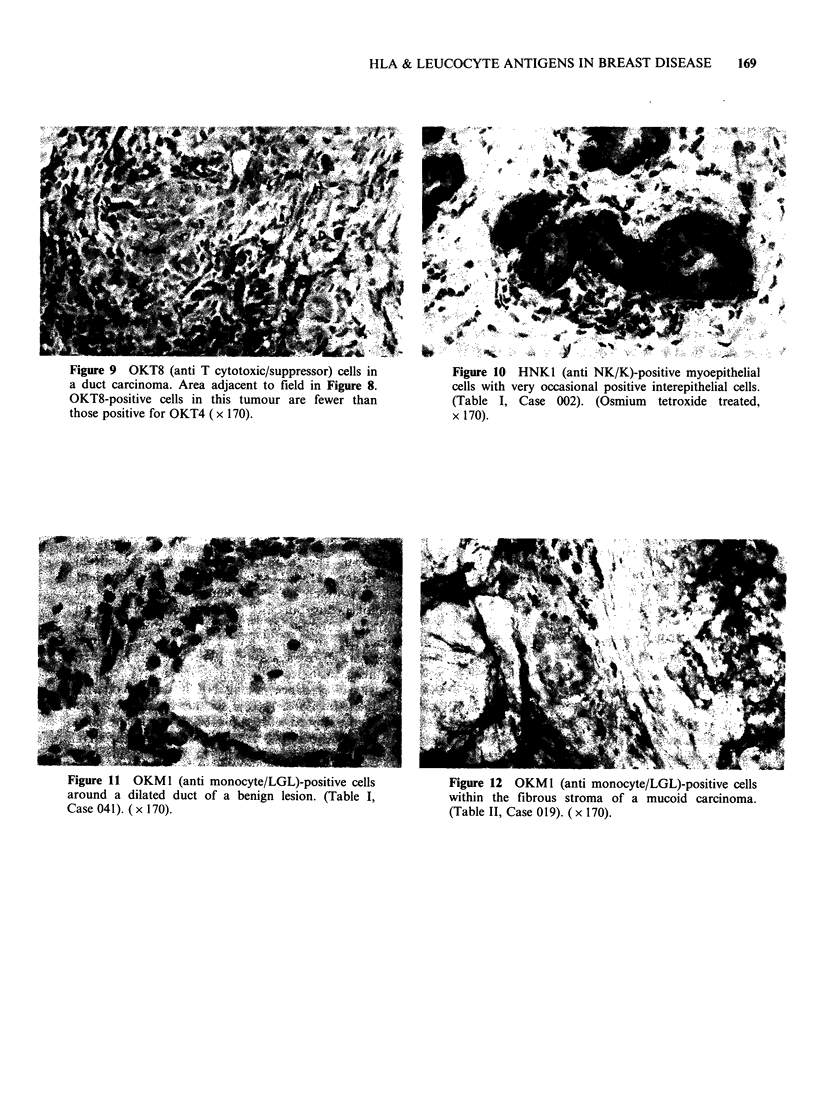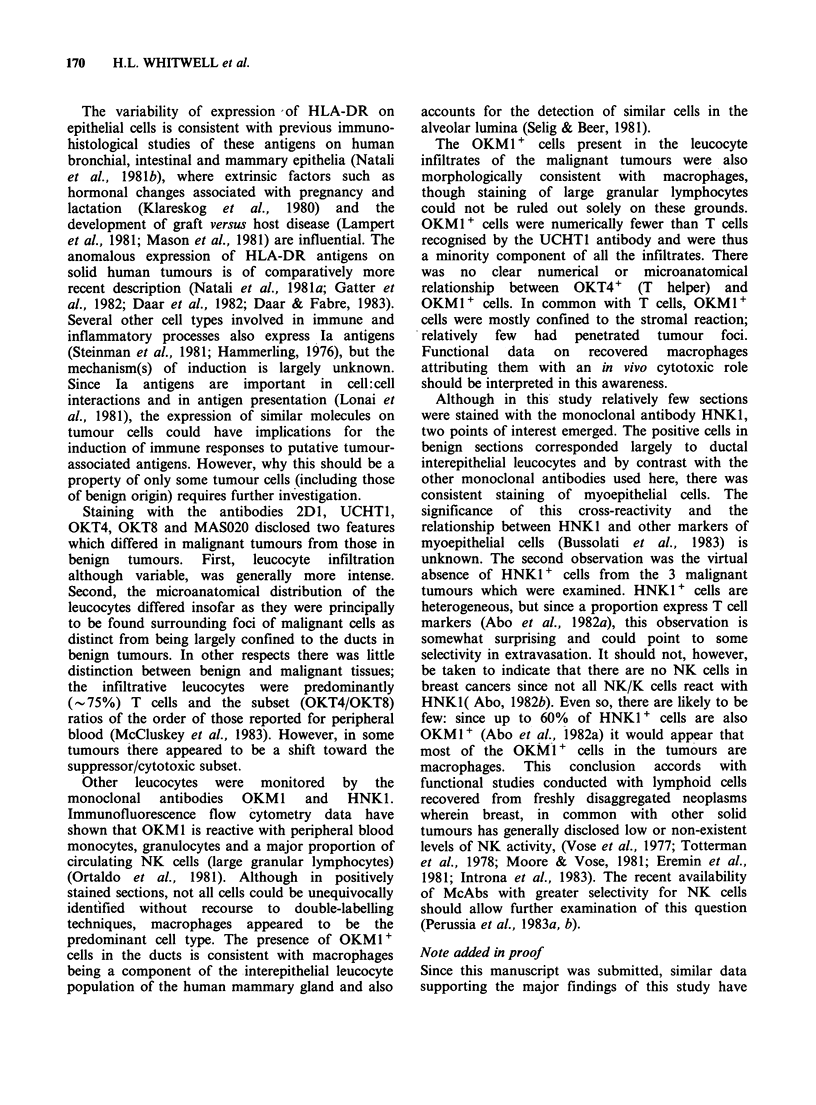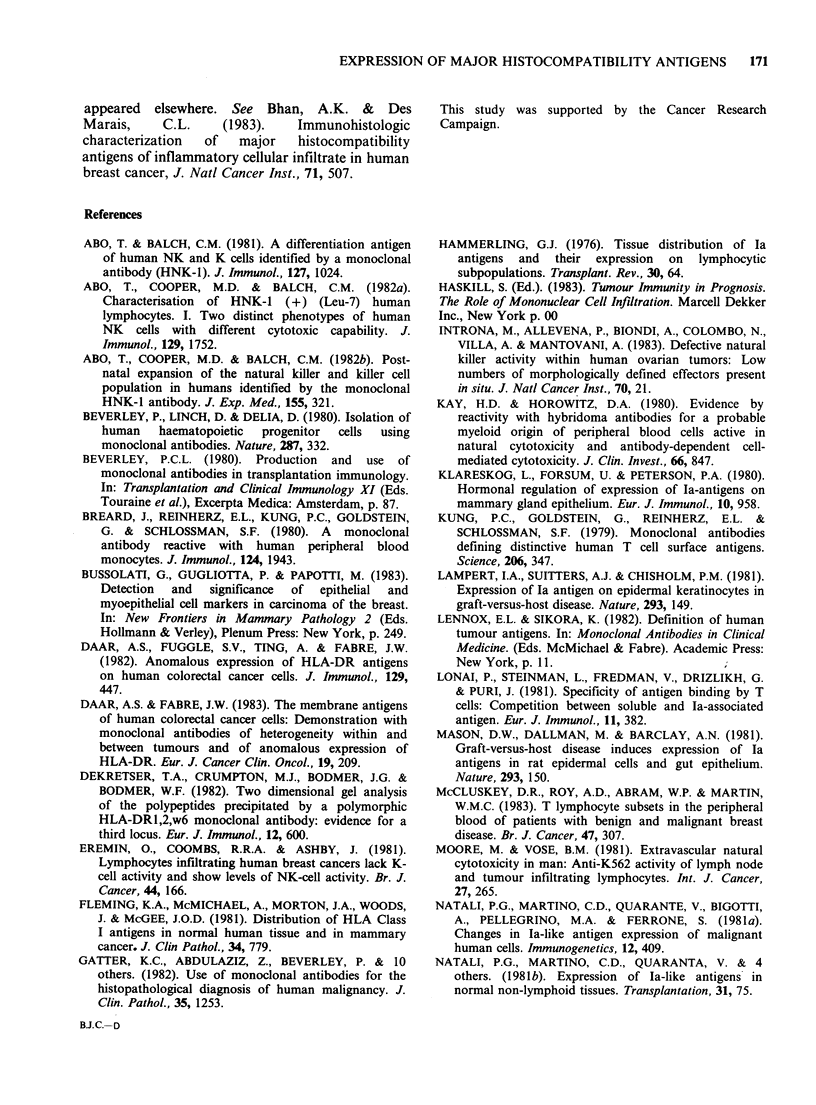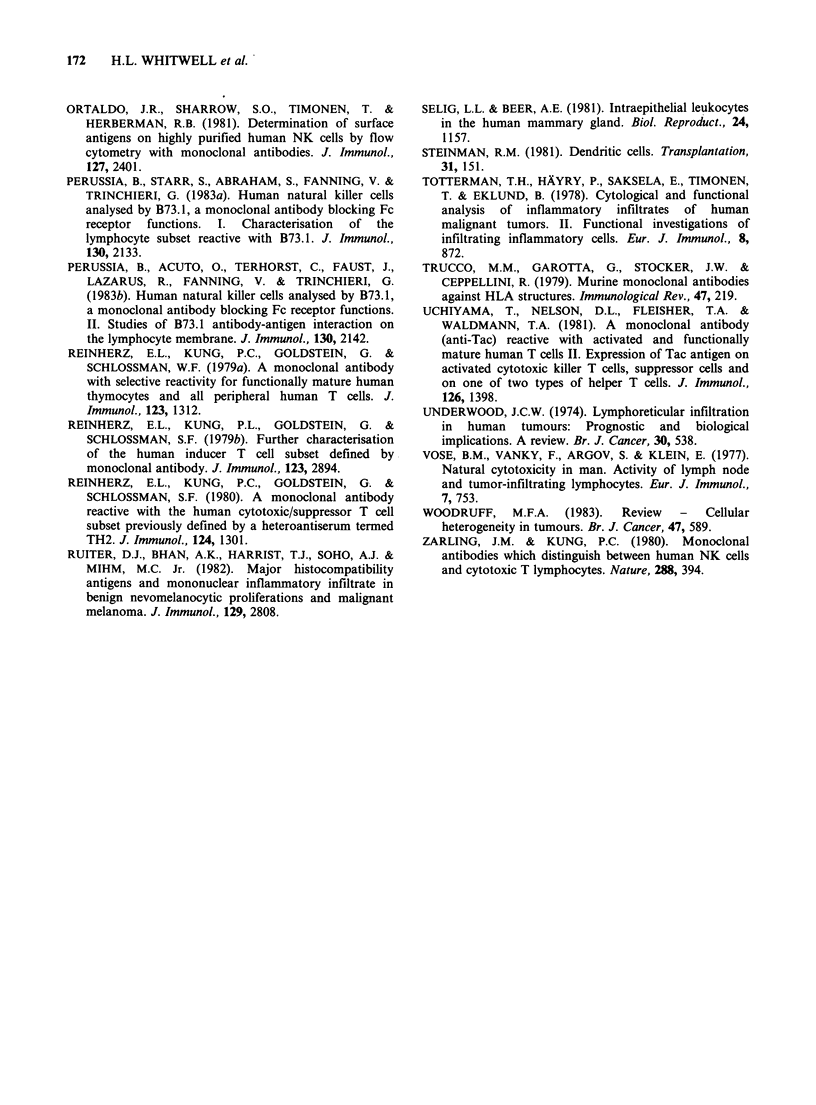# Expression of major histocompatibility antigens and leucocyte infiltration in benign and malignant human breast disease.

**DOI:** 10.1038/bjc.1984.28

**Published:** 1984-02

**Authors:** H. L. Whitwell, H. P. Hughes, M. Moore, A. Ahmed

## Abstract

**Images:**


*Br. J. Cancer* (1984), **49**, 161–172

### Expression of major histocompatibility antigens and leucocyte infiltration in benign and malignant human breast disease

H.L. Whitwell[1,2], H.P.A. Hughes[1]*, M. Moore[1] & A. Ahmed[2]

[1]*Department of Immunology, Paterson Laboratories, Christie Hospital and Holt Radium Institute, Manchester M20 9BX.* [2]*Department of Pathology, University of Manchester Medical School, Manchester M13 9PL.*

**Summary** The reactivity of murine monoclonal antibodies (McAbs) directed against the monomorphic determinants of Class I and Class II antigens of the major histocompatibility complex (MHC), and against antigens expressed by discrete populations of leucocytes was studied using the indirect immunoperoxidase technique on serial tissue sections of 16 benign and 17 malignant primary human breast tumours. Class I antigens (detected by the McAb 2A1) were consistently associated with stromal leucocytes, fibroblasts and vascular endothelium, but expression on epithelial cells particularly of malignant provenance, was more variable. Class II antigens (detected by TDR 31.1) were present upon a variety of cell types which also included sporadic expression on malignant and benign epithelia. The distribution of leucocytes grossly monitored with 2D1 (reactive with a common leucocyte antigen) was largely interepithelial and periductal in benign lesions. Leucocytes were generally more numerous in malignant tumours, where they were largely confined to the stroma. The majority ($\sim$75%) of leucocytes were T lymphocytes (reactive with UCHT1), some of which appeared to react with TDR 31.1 and were therefore activated. Ratios of helper/inducer ($OKT4^+$) and suppressor/cytotoxic ($OKT8^+$) subsets generally exceeded unity in malignant neoplasms. There was no correlation between the extent and distribution of T cells and the HLA status of the epithelial cells. Leucocytes detected by the monoclonal antibody OKM1 which reacts with monocytes/macrophages, granulocytes and large granular lymphocytes were numerically few and again mainly confined to the stroma. In a limited number of tests, leucocytes detected with HNK1, reactive with a differentiation antigen expressed on some cells which mediate natural and antibody-dependent cellular cytotoxicity *in vitro* although detectable interepithelially in benign tumours, were virtually absent from malignant tissue. HNK1 also cross-reacted with myoepithelial cells in the ducts of benign lesions.

Human malignant neoplasms not infrequently reveal mononuclear cell infiltrates which have features in common with immune-associated inflammatory reactions (Underwood, 1974). Although there is limited evidence for tumour-related functional activity in the infiltrating leucocyte compartment (see Haskill, 1983), the relationship of the phenomenon at a functional or histological level to biological behaviour remains largely obscure.

The majority of solid human neoplasms are characterised by marked cellular heterogeneity and any provisional assignment of *in situ* function will require the elucidation of the heterogeneity and microanatomical distribution of potential leucocytic effector cells relative to the tumour population. Such an analysis must also extend to the neoplastic compartment itself. Human tumour cells are phenotypically diverse (Woodruff, 1983) and although several useful markers exist (Lennox & Sikora, 1982) the antigens which evoke cellular immune responses have not been serologically defined.

The initiation of the present study coincided with reports of heterogeneity of expression of Class I (Fleming *et al.*, 1981) and anomalous expression of Class II (DR) antigens (Natali *et al.*, 1981*a*; Daar *et al.*, 1982) of the major histocompatibility complex (MHC) on primary human tumours. Since these MHC products have a crucial role in cell:cell interactions involving both the inductive and effector phases of the immune response it seemed reasonable to examine, as a preliminary approach, the HLA status of primary tumours in conjunction with leucocyte infiltration. For this purpose we have deployed the indirect immunoperoxidase technique using monoclonal antibodies (McAbs) against the major leucocyte populations and their subsets and McAbs to the monomorphic determinants of the HLA Class I and Class II antigens. Breast was the tissue of choice on account of the availability of benign and malignant tissue from the same organ and the absence of infection.

*Present address: Department of Immunology, St Georges Hospital Medical School, Cranmer Terrace, Tooting, London SW19.

Correspondence: H.L. Whitwell

Received 16 July 1983; accepted 26 October 1983.

© The Macmillan Press Ltd., 1984

We report here that the T lymphocyte-predominant leucocyte infiltrates of malignant tumours are quantitatively greater than those of benign neoplasms, there is no simple correlation with the expression of either MHC Class I or Class II antigens on the tumour cells; that the lymphocytic reaction, comprising T-helper and T-suppressor cells, usually in order of their frequency in peripheral blood, is confined mainly to the stroma and that the numbers of *potential* effector cells of whatever provenance (lymphoid or myelomonocytic) within the tumour mass are small.

### Patients and methods

### *Patients*

The patients were all admitted for excision/frozen section of a breast lump. Diagnosis was made on the frozen section and in all but one of the malignant cases mastectomy with or without axillary lymph clearance was carried out. Histological diagnosis was confirmed on paraffin sections.

Of the patients with benign breast lesions the age range was 22–65 years. Two patients (050 and 048) were taking oral contraceptives at the time of operation; none of the others were taking steroidal or anti-inflammatory drugs. In the malignant group the age range was 50–78 years.

One patient (046) had had a previous left mastectomy for carcinoma of the breast 6 years before with radiotherapy (left side only) and stilboestrol therapy. At the time of the right mastectomy (for a histologically similar tumour) the patient was taking tamoxifen only.

One patient (049) had a history of radiotherapy to the genito-urinary tract 15 years previously for an unknown reason.

One patient (022) with longstanding rheumatoid arthritis was taking cimetidine and ketoprofen, a non-steroidal anti-inflammatory agent.

### *Tissue specimens*

Firm tissue from the tumour mass (mastectomy specimens and excision biopsies) was wrapped in tin foil, snap frozen in liquid nitrogen and stored at −70°C or over liquid nitrogen. Serial sections 5–10 μm thick (depending on the properties of the section) were cut, dried at 37°C for 30 min and stored at −20°C under desiccated conditions prior to examination within 7 days. Prolonged storage at −20°C appeared to have detrimental effects on certain antigens, for example, those recognised by the OKT4 and HNK-1 antibodies.

### *Immunohistochemical staining*

Sections were fixed in acetone at room temperature for 5 min, air-dried and immersed in 20% newborn calf serum (NBCS, Flow Labs.) in Tris-HCl buffered saline (TBS; pH7.5, 0.05M Tris; 0.85% NaCl). Excess buffered NBCS was wiped away from the sections and they were incubated in the monoclonal first layer (TBS without McAb on control sections) for 60 min at 37°C in 100% relative humidity. Excess antibody was drained and the sections were washed for 3 × 5 min in TBS. They were then incubated in 1/250 dilution of horse radish peroxidase conjugated rabbit anti-mouse IgG (Dako) in TBS containing 6.6% normal human serum. Following 60 min incubation (37°C, 100% relative humidity) and 3 × 5 min washes in TBS, sections were incubated with 60 mg% diaminobenzidine. Immediately before use, the diaminobenzidine solution was filtered and 60 μl $H_2O_2$/100 ml substrate was added. Following incubation for 5–10 min sections were washed in TBS. The staining of those incubated with OKT4 or HNK-1 was enhanced by immersion in 1% phosphate-buffered osmium tetroxide for 2 min and subsequent washing in TBS. This did not affect the specificity of the reactions, but helped to visualise those cells the staining of which otherwise tended to be weak. Consequently, a more accurate assessment of the distribution of $HNK1^+$ cells and of T4/T8 ratios could be made. The sections were then washed in distilled water, counterstained for 5 sec, in Gill's no. 2 Haemalum, blued in hot tap water, dehydrated in a series of graded alcohols (50, 70, 95, and 100%) and cleared in xylene. Permanent mounts were made in Styrolyte (Raymond Lamb). The specificity of the antibodies was routinely controlled on sections of palatine tonsils. Staining in tissue sections treated with diaminobenzidine and hydrogen peroxide alone was routinely negligible in breast tissue.

### *Monoclonal antibodies*

The following murine McAbs were used in this study:

*2A1* IgG1 antibody identifying human HLA Class I (HLA-A, B, C) non-polymorphic determinant (Beverley, 1980).

*TDR31.1* IgG1 antibody recognising human HLA Class II (HLA-DR) monomorphic determinant (DeKretser *et al.*, 1982). This reagent was supplied as purified culture supernatant (400 μg $ml^{-1}$) and used at 1/200 dilution.

*MASO20* IgG1 antibody against human B cells (Clone 5/11 HLK) recognising a site close to the monomorphic determinant of the DR molecule (Trucco *et al.*, 1979). This antibody was not identical to TDR 31.1.

*2DI* IgG1 antibody directed against a human haemopoietic cell antigen (HLeI) of mol.wt. 70K dalton present on lymphoid and myeloid cells, weakly expressed on granulocytes, monocytes and early erythroid precursors. Absent from a wide variety of epithelia (Beverley *et al.*, 1980).

*UCHT1* IgG1 antibody reactive with antigen of 19K dalton mol.wt. expressed on peripheral T cells and showing identical reactivity with the monoclonal antibody OKT3 (Kung *et al.*, 1979).

*OKT4* IgG2b antibody reactive with 62K dalton mol.wt. antigen expressed on human T cells of helper/inducer subclass ($TH_2-T$ cells) (Kung *et al.*, 1979; Reinherz *et al.*, 1979*a*, *b*).

*OKT8* IgG2a antibody reactive with an undefined antigen of peripheral T cells of cytotoxic/suppressor subclass ($TH_2+$) (Reinherz *et al.*, 1980).

*OKM1* IgG2b antibody reactive with mature monocytes, granulocytes and certain circulating null cells (Breard *et al.*, 1980) including cells which mediate NK activity (Zarling & Kung, 1980) and antibody-dependent cellular cytotoxicity (Kay & Horowitz, 1980).

*HNK-1* IgM antibody (Leu 7) which defines a differentiation antigen selectively expressed on NK and antibody-dependent killer (K) cells (Abo & Balch, 1981).

TDR31.1 was a gift of Drs W. and J. Bodmer, Imperial Cancer Research Fund, London and 2A1, 2D1 and UCHT1, generous gifts of Dr P.C.L. Beverley, ICRF Human Tumour Immunology Unit, University College Hospital, London. OKT4, OKT8 and OKM1 were supplied by Ortho Pharmaceutical Corporation, New Jersey, USA; MAS020 by Sera-Lab, UK and HNK-1 by Becton Dickinson Monoclonal Center, Inc., USA.

### Results

Histopathological and immunocytochemical data on sections from the 16 benign and 17 malignant tissues are summarised in Tables I and II.

*2A1 (anti HLA, -A, -B, -C)*

Antigens recognised by this antibody which stained both the membrane and cytoplasm were detected consistently on the leucocytes, as well as endothelial cells and stromal fibroblasts of tumour tissue, but some epithelial cells of benign and malignant tissue were negative while others were positive with various degrees of staining intensity (Figure 1). In 5/16 benign tissue specimens staining was uniform but in the remaining 11 it was more variable. However, by contrast with the malignant tumours none of the benign lesions exhibited *completely* negative staining of epithelial cells with this McAb.

Uniform staining of epithelial cells was not observed in any of the 17 malignant tissue specimens. In 8, the pattern was heterogeneous (e.g. Figure 2) while the remainder were uniformly negative. There was no correlation between 2A1 and TDR31.1, 2D1 or UCHT1 staining.

*TDR31.1 (anti HLA-DR)*

Reactivity with antibody ranged from weak, intermittent to strong membrane and cytoplasmic staining (Figure 3) of both the cells of benign and malignant tissues.

Lymphoid and non-lymphoid cells appeared to be stained, the latter comprising a range of morphological features including benign and malignant epithelial cells, elongated cells reminiscent of dendritic cells, endothelial cells in capillaries and macrophages. The staining of epithelial cells, benign and malignant – was sporadic and never uniform.

There was no correlation between epithelial TDR31.1 and 2D1 (or UCHT1) staining. However, lymphoid cells stained with TDR31.1 although numerically fewer were positively correlated with those stained with 2D1 and UCHT1 (Tables I and II).

*2D1 (anti leucocyte)*

Epithelial cells in all specimens were negative for antigens detected by this antibody. The majority of positive cells possessed the rounded morphology characteristic of lymphoid cells but occasional elongated cells mostly resembling fibroblasts in the periductular stroma were also stained. Round cell staining was mainly confined to the inter-epithelial and periductal areas in benign tissues, while elongated cells were most frequently found in the stroma (Figure 4).

Round cell staining was a more frequent and prominent feature of malignant than benign tissue and not, by contrast with the latter, principally found in association with ducts. Positive cells were characteristically found in the stroma surrounding tumour foci, with relatively few actually detectable within the tumour mass (Figure 5). There appeared to be no correlation between the extent and distribution of 2DI staining and necrosis.

*UCHT1 (anti pan T cell)*

Staining patterns observed with UCHT1 were

**Table I** Summary of immunohistological data derived from serial sections of 16 benign breast tumours

| Patient no. | Age | Histology | Epithelial Cell staining[b] 2A1 | Epithelial Cell staining[b] TDR31.1 | Inflammatory cell infiltrate staining[a] TDR31.1 | 2D1 | UCHT1 | MASO20 | OKT4/8 ratio | OKM1 |
|---|---|---|---|---|---|---|---|---|---|---|
| 002 | 37 | Adenosis | +/− | +/− | + | ++ | ++ | ++ | >1 | − |
| 048 | 22 | Adenosis | + | +/− | ++ | ++ | ++ | + | >1 | + |
| 031 | 49 | Adenosis | + | +/− | ++ | +++ | ++ | − | >1 | − |
| 047 | 32 | Adenosis | + | +/− | ++ | ++ | + | ++ | ND | − |
| 006 | 32 | Adenosis/fibrosis | +/− | +/− | + | ++ | ++ | + | >1 | − |
| 013 | 33 | Adenosis/fibrosis | +/− | +/− | ++ | +++ | ++ | + | >1 | + |
| 014 | 51 | Adenosis/fibrosis | +/− | +/− | ++ | ++ | + | + | >1 | + |
| 041 | 45 | Adenosis, fibrosis, duct ectasia | +/− | −/+ | ++ | ++ | ++ | + | >1 | + |
| 007 | 65 | Mild fibrosis | +/− | −/+ | + | + | + | − | >1 | − |
| 042 | 49 | Cystic mastopathy | +/− | −/+ | + | +++ | ++ | + | <1 | − |
| 023 | 44 | Sclerosing adenosis | +/− | −/+ | ++ | ++ | + | − | <1 | + |
| 036 | 43 | Adenosis/epitheliosis | +/− | +/− | ++ | ++ | ++ | + | >1 | + |
| 020 | 59 | Duct ectasia, epitheliosis, | + | +/− | +++ | +++ | ++ | ++ | >1 | + |
| 035 | 43 | Fibroadenoma | +/− | +/− | + | +++ | ++ | ++ | ND | − |
| 045 | 32 | Fibroadenoma | + | +/− | ++ | ++ | ++ | + | <1 | + |
| 050 | 27 | Fibroadenoma | +/− | +/− | ++ | +++ | ++ | + | >1 | + |

ND = Not Done.
[a]Staining reactions are scored on a semi-quantitative scale from 4+ (many cells stained) to − (no cells stained) for the inflammatory cell infiltrate, apart from OKM1 where the relative scale is from + to −.
[b]Staining reactions with anti-HLA antibodies are denoted by + (homogeneous staining); − (no staining) and +/− (heterogeneous staining).

similar in microanatomical distribution to those of 2D1 (Figures 6 and 7) such that UCHT1$^+$ cells accounted for ~75% 2D1$^+$ cells, regardless of the tissue examined. Some positive cells were noticeably larger than others and possibly corresponded to T cell blasts. 2D1 and UCHT1 staining was broadly correlated with lymphoid TDR31.1 staining (Tables I and II). Virtually none of the UCHT1$^+$ cells in the few malignant tumours also examined with HNK-1 (see below) appeared to react with the latter antibody, although certain similarities in the patterns of staining were observed in some benign lesions.

*OKT4/OKT8 (anti T helper/inducer/T suppressor/cytotoxic subsets*

OKT4/OKT8 ratios exceeded unity in the majority of benign (11/14) and malignant (12/17) tissues (e.g. Figures 8 & 9), and there was no evidence of microanatomic segregation, the pattern of distribution following that of UCHT1$^+$ cells in both benign and malignant tissues.

*MAS020 (anti B cell)*

Relatively few cells were stained with this McAb. Benign tissues were mainly characterised by the presence of small numbers of positive interepithelial cells. In the periductal areas and in stromal tissue, occasional staining was observed which appeared to correspond to fibroblasts. Epithelial cells were negative but a proportion of endothelial cells in some sections were positive. A feature in common with OKM1 was the staining of intraluminal material.

*HNK1 (anti NK/K cells)*

The numbers of specimens stained with this antibody were limited. No positive cells were observed in 3 malignant tissues, two of which – 003 and 009 – had marked leucocyte infiltrates. Tissues positive for OKM1 (003 and 010) were negative for HNK1. Two of 5 benign tissues (cases 002 and 014) gave positive reactions essentially confined to the ductal and periductal regions as described for UCHT1, 2D1 and OKM1 but to a quantitatively lesser extent. Of considerable interest was the reactivity, characteristic of this antibody, observed against myoepithelial cells (Figure 10) including their cytoplasmic processes.

*OKM1 (anti monocyte/large granular lymphocyte)*

Relative to 2D1, cells reactive with this antibody were generally fewer in all sections examined. In benign tissues, OKM1$^+$ cells with the morphology

**Table II** Summary of immunohistological data derived from serial sections of 17 malignant breast tumours

| Patient no. | Age | Histology | Other features | Epithelial cell staining 2A1 | Epithelial cell staining TDR31.1 | Inflammatory cell infiltrate staining TDR31.1 | 2D1 | UCHT1 | MAS020 | OKT4/8 ratio | OKM1 |
|---|---|---|---|---|---|---|---|---|---|---|---|
| 009 | 75 | Infiltrating lobular carcinoma | | – | – | +++ | +++ | +++ | ++ | <1 | – |
| 046 | 61 | Infiltrating and *in situ* lobular carcinoma | previous mastectomy (see text) | – | – | +++ | +++ | ++ | ++ | >1 | + |
| 003 | 59 | Infiltrating lobular carcinoma | | – | – | ++ | +++ | ++ | ++ | >1 | + |
| 019 | 67 | Mucoid carcinoma | necrosis | – | – | ++ | +++ | +++ | + | >1 | + |
| 040 | 52 | Infiltrating lobular carcinoma infiltrating and *in situ* ductal carcinoma | necrosis | +/– | +/– | ++ | ++++ | +++ | + | >1 | + |
| 022 | 52 | Intraduct carcinoma | necrosis | – | – | + | +++ | + | ++ | >1 | – |
| 032 | 61 | Infiltrating ductal carcinoma | necrosis | – | – | ++ | ++ | ++ | + | <1 | + |
| 053 | 51 | Infiltrating ductal carcinoma | | – | – | +++ | ++ | + | ++ | >1 | – |
| 026 | 70 | Infiltrating ductal carcinoma | | – | – | ++ | ++ | ++ | + | <1 | – |
| 052 | 75 | Infiltrating ductal carcinoma | necrosis | +/– | – | +++ | ++++ | +++ | + | >1 | – |
| 043 | 50 | Infiltrating ductal carcinoma | necrosis | +/– | +/– | +++ | +++ | ++ | ++ | >1 | + |
| 039 | 60 | Infiltrating ductal carcinoma | necrosis | +/– | (+)/– | ++ | +++ | ++ | ++ | <1 | + |
| 049 | 60 | Infiltrating ductal carcinoma | necrosis (no mastectomy) | +/– | – | ++ | ++++ | +++ | ++ | >1 | + |
| 010 | 78 | Infiltrating ductal carcinoma | | – | – | ++ | + | + | + | >1 | + |
| 059 | 62 | Infiltrating ductal carcinoma | | +/– | – | +++ | +++ | + | + | ~1 | + |
| 021 | 50 | Infiltrating ductal carcinoma with lobular "cancerization" | necrosis | +/(–) | +/– | ++++ | ++++ | ++++ | ++ | >1 | + |
| 033 | 55 | Infiltrating ductal carcinoma with lobular "cancerization" | | +/– | +/– | +++ | ++++ | +++ | + | >1 | – |

Staining reactions are scored as described for **Table I**.

of macrophages were most obvious in and around dilated ducts (Figure 11). In malignant tumours, staining from virtually none at all to clusters of cells again resembling macrophages were almost invariably confined to the stroma (Figure 12). A further feature of this McAb was staining of intraluminal cells (? macrophages) and acellular material in ducts of benign lesions (cf. MAS020) though not all ducts in a given section were necessarily positive in this respect.

### Discussion

The identification and characterisation of the various cell types involved at the host:tumour interface should ideally be carried out *in situ* under conditions where the structural integrity of the tissue is retained. Only in these circumstances can the inter-relationship between diverse cell populations be properly observed. The problem then becomes how to assign function into literally static milieu of interacting cells. Hitherto, the identification of immune cells in tissue sections has been largely limited to morphology but the recent availability of McAbs against different lymphocyte subsets offers the potential to interpret histological data in functional terms. Likewise, McAbs reactive with tumour cell membrane components may provide some insight into the extent of tumour heterogeneity and in the case of products of the MHC, have important implications for cell–cell interactions in the inductive and effector phases of the immune response.

Notwithstanding such advances, studies of this type are not without interpretative difficulties. Apart from being limited to a single time point in the natural history of the disease, the subdivision of specimens to meet the requirements of diagnostic histopathology, may introduce unavoidable sampling errors. Although reproducibility was within acceptable limits on serial sections from the same portion of tumour, there was virtually no opportunity for a direct comparison of the centre *versus* periphery.

A further problem is the identification of cells in circumstances where a determinant recognised by a given antibody is expressed by several cell types which cannot be unequivocally distinguished solely on morphological grounds. Although interpretation is to some extent assisted by the availability of other more definitive markers for cells of a given lineage, the distinction between some cell types requires the application of double-labelling techniques, which were beyond the scope of the present study.

While consistent staining of leucocytes and stromal cells was obtained with the anti-HLA-A, -B, -C, McAb (2A1), reactivity with tumour cells particularly of malignant provenance was less predictable. Focal staining indicative of heterogeneity of Class I antigen expression characterised many sections – malignant and benign and approximately one-half of the former were negative for this antibody under conditions where adjacent stromal tissue was strongly positive. In this respect our data are similar to those of Fleming *et al.* (1981) who using immunofluorescence with the McAb PA 2.6 to HLA Class I antigens, reported a high detection rate of these molecules in the ductal epithelium of non-malignant breast tissue, but marked heterogeneity in the epithelium of malignant tumours.

Selective absorption from plasma of HLA Class I antigens on to the surface of epithelial cells, or masking by circulating antibody to Class I molecules are not adequate explanations of the inter- and intra-tumour variation observed with 2A1, although subtle undefined conformational changes in the monomorphic determinant which might render it unreactive with the monoclonal antibody could conceivably occur. The data rather suggest that the expression of HLA Class I molecules in a major group of primary malignant breast tumours is reduced.

The reduction of HLA Class I antigens or their heterogeneous distribution within a neoplasm may have important biological implications e.g. for the associative recognition of tumour antigens by T cells and hence for immunosurveillance.

However, there was no apparent qualitative or quantitative correlation between leucocyte infiltration and HLA Class I antigen expression. In this report our data contrast with recent experience with dysplastic and malignant nevomelanocytes where the degree of mononuclear cell infiltration correlated with the expression of HLA (or $\beta_2$ microglobulin) on nevomelanocytes (Ruiter *et al.*, 1982).

Staining with the anti HLA-DR McAb (TDR31.1) also comprised lymphoid and non-lymphoid cells. The numbers of leucocytes stained were usually less than those stained with either 2D1 or UCHT1, suggesting that not all the tumour-associated T cells were $DR^+$. However, the extent of T cell activation by this criterion alone is difficult to assess since an anti HLA-DR McAb would also be expected to strain the minority ($\sim$25% leucocytes) B cell and monocyte/macrophage populations. The deployment of the Tac monoclonal antibody to the IL-2 receptor on activated T cells (Uchiyama *et al.*, 1981) might resolve the issue.

**Figure 1** 2A1 (anti HLA-A, -B, -C)-positive interepithelial leucocytes and periductal cells. Epithelial cell staining is variable. (Table I, Case 006, adenosis). (×170).

**Figure 2** 2A1 (anti HLA-A, -B, -C)-positive cells in an infiltrating ductal carcinoma (Table II, Case 021). The heterogeneous staining pattern is typical of approx. 50% of the malignant tumours in this series. (×170).

**Figure 3** TDR31.1 (anti HLA-DR)-positive epithelial and periductular cells. (Table I, Case 013, adenosis/fibrosis). Note the variation in staining intensity among the ducts. (×170).

**Figure 4** 2D1 (anti common leucocyte)-positive interepithelial and periductal cells. Epithelial cells are negative. (Table I, Case 002). (×150).

**Figure 5** 2D1 (anti common leucocyte)-positive round cells surrounding a duct carcinoma. (Table II, Case 021). (×70).

**Figure 6** UCHT1 (anti pan T cell)-positive inter-epithelial and periductal cells. (Table I, Case 002, adenosis). (×170).

**Figure 7** UCHT1 (anti pan T cell)-positive round cells surrounding duct carcinoma cells. (Table II, Case 021). (×170).

**Figure 8** Preponderance of OKT4 (anti T helper)-positive cells in a duct carcinoma (Table II, Case 021). (Osmium tetroxide treated, ×170).

**Figure 9** OKT8 (anti T cytotoxic/suppressor) cells in a duct carcinoma. Area adjacent to field in **Figure 8**. OKT8-positive cells in this tumour are fewer than those positive for OKT4 (×170).

**Figure 10** HNK1 (anti NK/K)-positive myoepithelial cells with very occasional positive interepithelial cells. (Table I, Case 002). (Osmium tetroxide treated, ×170).

**Figure 11** OKM1 (anti monocyte/LGL)-positive cells around a dilated duct of a benign lesion. (Table I, Case 041). (×170).

**Figure 12** OKM1 (anti monocyte/LGL)-positive cells within the fibrous stroma of a mucoid carcinoma. (Table II, Case 019). (×170).

The variability of expression of HLA-DR on epithelial cells is consistent with previous immunohistological studies of these antigens on human bronchial, intestinal and mammary epithelia (Natali *et al.*, 1981*b*), where extrinsic factors such as hormonal changes associated with pregnancy and lactation (Klareskog *et al.*, 1980) and the development of graft *versus* host disease (Lampert *et al.*, 1981; Mason *et al.*, 1981) are influential. The anomalous expression of HLA-DR antigens on solid human tumours is of comparatively more recent description (Natali *et al.*, 1981*a*; Gatter *et al.*, 1982; Daar *et al.*, 1982; Daar & Fabre, 1983). Several other cell types involved in immune and inflammatory processes also express Ia antigens (Steinman *et al.*, 1981; Hammerling, 1976), but the mechanism(s) of induction is largely unknown. Since Ia antigens are important in cell:cell interactions and in antigen presentation (Lonai *et al.*, 1981), the expression of similar molecules on tumour cells could have implications for the induction of immune responses to putative tumour-associated antigens. However, why this should be a property of only some tumour cells (including those of benign origin) requires further investigation.

Staining with the antibodies 2D1, UCHT1, OKT4, OKT8 and MAS020 disclosed two features which differed in malignant tumours from those in benign tumours. First, leucocyte infiltration although variable, was generally more intense. Second, the microanatomical distribution of the leucocytes differed insofar as they were principally to be found surrounding foci of malignant cells as distinct from being largely confined to the ducts in benign tumours. In other respects there was little distinction between benign and malignant tissues; the infiltrative leucocytes were predominantly ($\sim$75%) T cells and the subset (OKT4/OKT8) ratios of the order of those reported for peripheral blood (McCluskey *et al.*, 1983). However, in some tumours there appeared to be a shift toward the suppressor/cytotoxic subset.

Other leucocytes were monitored by the monoclonal antibodies OKM1 and HNK1. Immunofluorescence flow cytometry data have shown that OKM1 is reactive with peripheral blood monocytes, granulocytes and a major proportion of circulating NK cells (large granular lymphocytes) (Ortaldo *et al.*, 1981). Although in positively stained sections, not all cells could be unequivocally identified without recourse to double-labelling techniques, macrophages appeared to be the predominant cell type. The presence of OKM1$^+$ cells in the ducts is consistent with macrophages being a component of the interepithelial leucocyte population of the human mammary gland and also accounts for the detection of similar cells in the alveolar lumina (Selig & Beer, 1981).

The OKM1$^+$ cells present in the leucocyte infiltrates of the malignant tumours were also morphologically consistent with macrophages, though staining of large granular lymphocytes could not be ruled out solely on these grounds. OKM1$^+$ cells were numerically fewer than T cells recognised by the UCHT1 antibody and were thus a minority component of all the infiltrates. There was no clear numerical or microanatomical relationship between OKT4$^+$ (T helper) and OKM1$^+$ cells. In common with T cells, OKM1$^+$ cells were mostly confined to the stromal reaction; relatively few had penetrated tumour foci. Functional data on recovered macrophages attributing them with an *in vivo* cytotoxic role should be interpreted in this awareness.

Although in this study relatively few sections were stained with the monoclonal antibody HNK1, two points of interest emerged. The positive cells in benign sections corresponded largely to ductal interepithelial leucocytes and by contrast with the other monoclonal antibodies used here, there was consistent staining of myoepithelial cells. The significance of this cross-reactivity and the relationship between HNK1 and other markers of myoepithelial cells (Bussolati *et al.*, 1983) is unknown. The second observation was the virtual absence of HNK1$^+$ cells from the 3 malignant tumours which were examined. HNK1$^+$ cells are heterogeneous, but since a proportion express T cell markers (Abo *et al.*, 1982*a*), this observation is somewhat surprising and could point to some selectivity in extravasation. It should not, however, be taken to indicate that there are no NK cells in breast cancers since not all NK/K cells react with HNK1( Abo, 1982*b*). Even so, there are likely to be few: since up to 60% of HNK1$^+$ cells are also OKM1$^+$ (Abo *et al.*, 1982a) it would appear that most of the OKM1$^+$ cells in the tumours are macrophages. This conclusion accords with functional studies conducted with lymphoid cells recovered from freshly disaggregated neoplasms wherein breast, in common with other solid tumours has generally disclosed low or non-existent levels of NK activity, (Vose *et al.*, 1977; Totterman *et al.*, 1978; Moore & Vose, 1981; Eremin *et al.*, 1981; Introna *et al.*, 1983). The recent availability of McAbs with greater selectivity for NK cells should allow further examination of this question (Perussia *et al.*, 1983*a*, *b*).

*Note added in proof*

Since this manuscript was submitted, similar data supporting the major findings of this study have appeared elsewhere. *See* Bhan, A.K. & Des Marais, C.L. (1983). Immunohistologic characterization of major histocompatibility antigens of inflammatory cellular infiltrate in human breast cancer, *J. Natl Cancer Inst.*, **71**, 507.

This study was supported by the Cancer Research Campaign.

### References

ABO, T. & BALCH, C.M. (1981). A differentiation antigen of human NK and K cells identified by a monoclonal antibody (HNK-1). *J. Immunol.*, **127**, 1024.

ABO, T., COOPER, M.D. & BALCH, C.M. (1982*a*). Characterisation of HNK-1 (+) (Leu-7) human lymphocytes. I. Two distinct phenotypes of human NK cells with different cytotoxic capability. *J. Immunol.*, **129**, 1752.

ABO, T., COOPER, M.D. & BALCH, C.M. (1982*b*). Postnatal expansion of the natural killer and killer cell population in humans identified by the monoclonal HNK-1 antibody. *J. Exp. Med.*, **155**, 321.

BEVERLEY, P., LINCH, D. & DELIA, D. (1980). Isolation of human haematopoietic progenitor cells using monoclonal antibodies. *Nature*, **287**, 332.

BEVERLEY, P.C.L. (1980). Production and use of monoclonal antibodies in transplantation immunology. In: *Transplantation and Clinical Immunology XI* (Eds. Touraine *et al.*), Excerpta Medica: Amsterdam, p. 87.

BREARD, J., REINHERZ, E.L., KUNG, P.C., GOLDSTEIN, G. & SCHLOSSMAN, S.F. (1980). A monoclonal antibody reactive with human peripheral blood monocytes. *J. Immunol.*, **124**, 1943.

BUSSOLATI, G., GUGLIOTTA, P. & PAPOTTI, M. (1983). Detection and significance of epithelial and myoepithelial cell markers in carcinoma of the breast. In: *New Frontiers in Mammary Pathology 2* (Eds. Hollmann & Verley), Plenum Press: New York, p. 249.

DAAR, A.S., FUGGLE, S.V., TING, A. & FABRE, J.W. (1982). Anomalous expression of HLA-DR antigens on human colorectal cancer cells. *J. Immunol.*, **129**, 447.

DAAR, A.S. & FABRE, J.W. (1983). The membrane antigens of human colorectal cancer cells: Demonstration with monoclonal antibodies of heterogeneity within and between tumours and of anomalous expression of HLA-DR. *Eur. J. Cancer Clin. Oncol.*, **19**, 209.

DEKRETSER, T.A., CRUMPTON, M.J., BODMER, J.G. & BODMER, W.F. (1982). Two dimensional gel analysis of the polypeptides precipitated by a polymorphic HLA-DR1,2,w6 monoclonal antibody: evidence for a third locus. *Eur. J. Immunol.*, **12**, 600.

EREMIN, O., COOMBS, R.R.A. & ASHBY, J. (1981). Lymphocytes infiltrating human breast cancers lack K-cell activity and show levels of NK-cell activity. *Br. J. Cancer*, **44**, 166.

FLEMING, K.A., McMICHAEL, A., MORTON, J.A., WOODS, J. & McGEE, J.O.D. (1981). Distribution of HLA Class I antigens in normal human tissue and in mammary cancer. *J. Clin Pathol.*, **34**, 779.

GATTER, K.C., ABDULAZIZ, Z., BEVERLEY, P. & 10 others. (1982). Use of monoclonal antibodies for the histopathological diagnosis of human malignancy. *J. Clin. Pathol.*, **35**, 1253.

HAMMERLING, G.J. (1976). Tissue distribution of Ia antigens and their expression on lymphocytic subpopulations. *Transplant. Rev.*, **30**, 64.

HASKILL, S. (Ed.). (1983). *Tumour Immunity in Prognosis. The Role of Mononuclear Cell Infiltration.* Marcell Dekker Inc., New York p. 00

INTRONA, M., ALLEVENA, P., BIONDI, A., COLOMBO, N., VILLA, A. & MANTOVANI, A. (1983). Defective natural killer activity within human ovarian tumors: Low numbers of morphologically defined effectors present *in situ*. *J. Natl Cancer Inst.*, **70**, 21.

KAY, H.D. & HOROWITZ, D.A. (1980). Evidence by reactivity with hybridoma antibodies for a probable myeloid origin of peripheral blood cells active in natural cytotoxicity and antibody-dependent cell-mediated cytotoxicity. *J. Clin. Invest.*, **66**, 847.

KLARESKOG, L., FORSUM, U. & PETERSON, P.A. (1980). Hormonal regulation of expression of Ia-antigens on mammary gland epithelium. *Eur. J. Immunol.*, **10**, 958.

KUNG, P.C., GOLDSTEIN, G., REINHERZ, E.L. & SCHLOSSMAN, S.F. (1979). Monoclonal antibodies defining distinctive human T cell surface antigens. *Science*, **206**, 347.

LAMPERT, I.A., SUITTERS, A.J. & CHISHOLM, P.M. (1981). Expression of Ia antigen on epidermal keratinocytes in graft-versus-host disease. *Nature*, **293**, 149.

LENNOX, E.L. & SIKORA, K. (1982). Definition of human tumour antigens. In: *Monoclonal Antibodies in Clinical Medicine*. (Eds. McMichael & Fabre). Academic Press: New York, p. 11.

LONAI, P., STEINMAN, L., FREDMAN, V., DRIZLIKH, G. & PURI, J. (1981). Specificity of antigen binding by T cells: Competition between soluble and Ia-associated antigen. *Eur. J. Immunol.*, **11**, 382.

MASON, D.W., DALLMAN, M. & BARCLAY, A.N. (1981). Graft-versus-host disease induces expression of Ia antigens in rat epidermal cells and gut epithelium. *Nature*, **293**, 150.

McCLUSKEY, D.R., ROY, A.D., ABRAM, W.P. & MARTIN, W.M.C. (1983). T lymphocyte subsets in the peripheral blood of patients with benign and malignant breast disease. *Br. J. Cancer*, **47**, 307.

MOORE, M. & VOSE, B.M. (1981). Extravascular natural cytotoxicity in man: Anti-K562 activity of lymph node and tumour infiltrating lymphocytes. *Int. J. Cancer*, **27**, 265.

NATALI, P.G., MARTINO, C.D., QUARANTE, V., BIGOTTI, A., PELLEGRINO, M.A. & FERRONE, S. (1981*a*). Changes in Ia-like antigen expression of malignant human cells. *Immunogenetics*, **12**, 409.

NATALI, P.G., MARTINO, C.D., QUARANTA, V. & 4 others. (1981*b*). Expression of Ia-like antigens in normal non-lymphoid tissues. *Transplantation*, **31**, 75.

ORTALDO, J.R., SHARROW, S.O., TIMONEN, T. & HERBERMAN, R.B. (1981). Determination of surface antigens on highly purified human NK cells by flow cytometry with monoclonal antibodies. *J. Immunol.*, **127**, 2401.

PERUSSIA, B., STARR, S., ABRAHAM, S., FANNING, V. & TRINCHIERI, G. (1983*a*). Human natural killer cells analysed by B73.1, a monoclonal antibody blocking Fc receptor functions. I. Characterisation of the lymphocyte subset reactive with B73.1. *J. Immunol.*, **130**, 2133.

PERUSSIA, B., ACUTO, O., TERHORST, C., FAUST, J., LAZARUS, R., FANNING, V. & TRINCHIERI, G. (1983*b*). Human natural killer cells analysed by B73.1, a monoclonal antibody blocking Fc receptor functions. II. Studies of B73.1 antibody-antigen interaction on the lymphocyte membrane. *J. Immunol.*, **130**, 2142.

REINHERZ, E.L., KUNG, P.C., GOLDSTEIN, G. & SCHLOSSMAN, W.F. (1979*a*). A monoclonal antibody with selective reactivity for functionally mature human thymocytes and all peripheral human T cells. *J. Immunol.*, **123**, 1312.

REINHERZ, E.L., KUNG, P.L., GOLDSTEIN, G. & SCHLOSSMAN, S.F. (1979*b*). Further characterisation of the human inducer T cell subset defined by monoclonal antibody. *J. Immunol.*, **123**, 2894.

REINHERZ, E.L., KUNG, P.C., GOLDSTEIN, G. & SCHLOSSMAN, S.F. (1980). A monoclonal antibody reactive with the human cytotoxic/suppressor T cell subset previously defined by a heteroantiserum termed TH2. *J. Immunol.*, **124**, 1301.

RUITER, D.J., BHAN, A.K., HARRIST, T.J., SOHO, A.J. & MIHM, M.C. Jr. (1982). Major histocompatibility antigens and mononuclear inflammatory infiltrate in benign nevomelanocytic proliferations and malignant melanoma. *J. Immunol.*, **129**, 2808.

SELIG, L.L. & BEER, A.E. (1981). Intraepithelial leukocytes in the human mammary gland. *Biol. Reproduct.*, **24**, 1157.

STEINMAN, R.M. (1981). Dendritic cells. *Transplantation*, **31**, 151.

TOTTERMAN, T.H., HÄYRY, P., SAKSELA, E., TIMONEN, T. & EKLUND, B. (1978). Cytological and functional analysis of inflammatory infiltrates of human malignant tumors. II. Functional investigations of infiltrating inflammatory cells. *Eur. J. Immunol.*, **8**, 872.

TRUCCO, M.M., GAROTTA, G., STOCKER, J.W. & CEPPELLINI, R. (1979). Murine monoclonal antibodies against HLA structures. *Immunological Rev.*, **47**, 219.

UCHIYAMA, T., NELSON, D.L., FLEISHER, T.A. & WALDMANN, T.A. (1981). A monoclonal antibody (anti-Tac) reactive with activated and functionally mature human T cells II. Expression of Tac antigen on activated cytotoxic killer T cells, suppressor cells and on one of two types of helper T cells. *J. Immunol.*, **126**, 1398.

UNDERWOOD, J.C.W. (1974). Lymphoreticular infiltration in human tumours: Prognostic and biological implications. A review. *Br. J. Cancer*, **30**, 538.

VOSE, B.M., VANKY, F., ARGOV, S. & KLEIN, E. (1977). Natural cytotoxicity in man. Activity of lymph node and tumor-infiltrating lymphocytes. *Eur. J. Immunol.*, **7**, 753.

WOODRUFF, M.F.A. (1983). Review – Cellular heterogeneity in tumours. *Br. J. Cancer*, **47**, 589.

ZARLING, J.M. & KUNG, P.C. (1980). Monoclonal antibodies which distinguish between human NK cells and cytotoxic T lymphocytes. *Nature*, **288**, 394.